# Effect of a digital-based integrated exercise and sleep intervention for older adults with depression: study protocol for a stepped-wedge cluster randomized controlled trial

**DOI:** 10.1186/s12877-026-07071-z

**Published:** 2026-02-02

**Authors:** Nan Zhang, Cui Wang, Yuling Ga, Kaoershaer Ailimu, Shanshan Chen, Miao Miao, Lu Zheng, Yajie Che, Lin Li, Mingjie Luo, Meng Zhao, Ping Yan, Mei Sun, Siyuan Tang

**Affiliations:** 1 School of Nursing, Xinjiang Medical University, Urumqi, Xinjiang China; 2 Xiangya School of Nursing, Central South University, Changsha, Hunan China; 3 School of Nursing, Zhejiang Chinese Medical University, Hangzhou, Zhejiang China; 4 Xinjiang Key Laboratory of Mental Development and Learning Science, School of Psychology, Xinjiang Normal University, Urumqi, Xinjiang China; 5 Department of Psychology, The First Affiliated Hospital of Xinjiang Medical University, Urumqi, Xinjiang China

**Keywords:** Depression, Older adults, Exercise, Sleep hygiene, Stepped-wedge cluster-randomized controlled trial, Study protocol

## Abstract

Geriatric depression affects 12.95-28.4% of adults aged ≥ 60, yet treatment rates remain critically low globally. Lifestyle factors, particularly exercise and sleep demonstrate therapeutic potential, integrated interventions may exert synergistic effects on geriatric depression, though such interventions remain scarce. The Geriatric Exercise-Sleep Optimization (GESO) project aims to evaluate the clinical efficacy and cost-effectiveness of a combined exercise and sleep health intervention in alleviating depressive symptoms among community-dwelling older adults with depression, and exploring the potential underlying mechanisms. This is a stepped-wedge cluster-randomized trial (SW-CRT). A 12-week integrated exercise and sleep intervention will be implemented to all eligible participants during the study period. The primary aim is to evaluate the clinical efficacy in alleviating depressive symptoms. Secondary aims are to evaluate the additional health outcomes (i.e., quality of life, physical activity level, daily step count, sleep quality, and anxiety symptom), cost-effectiveness, and potential mechanisms. Costs will be aggregated and analyzed for economic evaluation. Costs will be aggregated and analyzed for economic evaluation. Salivary measured BDNF and irisin levels, and EEG-based brain function connectivity will be collected to assess potential intervention mechanisms. Mixed-effect linear regression models will be used to evaluate the effects of the integrated exercise-sleep intervention on primary and secondary outcomes. This study is expected to provide an effective and practical mode for an integrated exercise and sleep intervention among community-dwelling older adults with depression. Intended outcomes of the trial will facilitate changes in best practice to improve outcomes for this population.

**Trial registration**

Chinese Clinical Trail Registry ChiCTR2500107641, Registration date: 15 August 2025.

**Supplementary Information:**

The online version contains supplementary material available at 10.1186/s12877-026-07071-z.

## Background

Geriatric depression is a worldwide public issue and is becoming more prevalent with population aging. Nearly 12.95% to 28.4% of people older than 60 years experience depression [1, 2], of whom 7.3% meet DSM-IV criteria of major depression (MDD), although only 0.5% are considered to receive adequate treatment [3]. Minimally adequate treatment (MAT) for major depression ranges from 23.0% in high-income countries to 3.0% in low- and lower middle-income countries [4]. This inadequate treatment coverage for geriatric depression remains a major barrier to older adults’ mental health and well-being.

Modifiable lifestyle factors, particularly physical activity and sleep quality, play a crucial role in maintaining physiological and psychological functions in older adults [5–7]. For instance, physical activity and sleep quality revealed significant main effects and significant interaction effect on cognitive function among older adults [8]. From the lifestyle psychiatry perspective, research has established that both the onset and symptoms of various mental disorders (i.e., depression) are also linked to modifiable lifestyle factors, such as physical activity and sleep [9]. Empirical research has demonstrated that behavioral interventions targeting sleep disturbances [10, 11] and physical activity/exercise exert benefits on depressive symptoms [12]. For adults with non-severe depression, exercise has shown no difference in effectiveness compared with pharmacological interventions [13]. Interventions aiming at promoting sleep, such as practices related to sleep hygiene, have demonstrated a positive impact on alleviating depression [14, 15]. In addition, exercise has also been shown to improve sleep quality in older adults [16], indicating that it may have synergistic positive effects with sleep on depression. Besides these, our previous study demonstrated an economic benefit from combined physical activity and sleep intervention for older adults with depression. Specifically, we employed a target trail emulation approach controlling both base and time-varying covariates and found that for older adults with depression, adopting regular exercise and adequate sleep could significantly reduce long-term healthcare expenditures and the risk of catastrophic healthcare expenditure. Practice guidelines for mood disorders indicate that actions involving lifestyle changes are foundational and essentially non-negotiable to all patients [17]. Additionally, in most regions, people with mental issues often experience psychological distress and are stigmatized [18], highlighting the importance of implementing interventions carrying potentially destigmatizing effects.

Muscle-brain crosstalk has emerged as a pivotal mechanism for explaining the antidepressant effects of exercise, particularly in the context of geriatric depression where somatic comorbidities like sarcopenia are prevalent [19, 20]. Central to this dialogue is the exercise-induced myokine irisin, which is cleaved from FNDC5, acts as a hormone on several distant targets, including the brain. That is, its ability to cross the blood-brain barrier positions it as a key mediator of exercise’s effects on the central nervous system [8, 21], which can likewise contribute to increased brain-derived neurotrophic factor levels. Critically, exercise-induced skeletal muscle contraction increases blood concentrations of peripheral factors, such as the brain-derived neurotrophic factor (BDNF) and irisin, which have been shown to improve sleep depth in animals [21, 22]. Furthermore, sleep homeostasis is often associated with stress-related mental disorders, including depression [23]. It is reasonable to infer that sleep improvement might directly or indirectly alleviate depression by regulating the BDNF levels. Additionally, while serum is the most commonly used sample for detecting irisin and BDNF, previous study suggested that both saliva and serum sample would represent a valid and less invasive method for irisin detection [24]. Saliva BDNF has also been established as a valid biomarker in mental health research [25, 26]. Therefore, quantifying these biomarkers in saliva is expected to provide crucial insights into the pathophysiological mechanisms underlying geriatric depression and the therapeutic potential of our integrated exercise-sleep intervention.

The major limitations of existing trials are as follows. First, previous studies have predominantly employed single-component interventions (exercise or sleep health promotion) rather than developing multi-component intervention packages, despite evidence showing that the integrated interventions combining physical activity and sleep health are effective and acceptable for older adults [7, 15]. Furthermore, socio-ecological factors have to be proved to influence the success of lifestyle interventions [27]. For instance, promoting sustainable exercise among older adults requires attention to contextual factors such as social support, accessible infrastructure, security, and social position. Therefore, a comprehensive intervention package should consider both the integration of core health behaviors and its cost-effectiveness. Second, the majority of studies rely heavily on subjective measures, making it difficult to elucidate the potential pathways of intervention effects More objective outcomes, such as those derived from accelerometry, electroencephalogram (EEG), and salivary biomarkers, should be incorporated to complement the outcome assessment framework. These multi-faceted measurements are collectively designed to elucidate both the effects of the intervention and their underlying mechanisms. Third, previous trials have generally included participants with above threshold-level depression (i.e., MDD) and have rarely focused exclusively on subthreshold depression. However, even subclinical depressive symptoms in older adults are associated with reduced quality of life and functional impairment [28, 29], underscoring the need for greater attention to this population.

Randomized controlled trials (RCTs) are considered as the most robust research design for establishing a cause-effect relationship between interventions and outcomes. Such studies have typically been conducted to evaluate the effectiveness of exercise or sleep interventions among community-dwelling older adults in prior studies [11, 14, 15, 30]. However, parallel RCTs may be impractical or unethical in certain circumstances. Furthermore, because the intervention group receives the intervention simultaneously, it cannot analyze the impact of intervention timing (i.e., intervening late versus intervening early) on outcomes. The stepped-wedge cluster-randomized trial (SW-CRT) is a variant of the cluster trial design which allows all clusters to sequentially undergo the intervention and act as their own controls during the study period [31, 32]. Thus, this design can determine whether the timing of the intervention impacts its effectiveness by exploring the effect of intervention sequence. The SW-CRT also ensures that more participating clusters are exposed to the intervention at the end of the study, thereby enabling researchers to control time effects and consequently improve the precision of the study. This is also a preferable design on ethical grounds because it deploys the transformation at all communities for stakeholders, rather than having some of them serve solely as controls” [33]. Furthermore, a SW-CRT design is particularly recommended for testing an intervention with strong evidence to yield beneficial effects or that is very unlikely to cause harm (i.e., exercise and sleep intervention for older adults) [32, 34], as is the case in our study. Another notable strength is that the SW-CRT design is the only feasible alternative, given that logistical, practical, or financial constraints prevent simultaneous intervention delivery to all participants. When it comes to specific areas, especially the study of sub-clinical or mild-to-moderate mental problems, the SW-CRT design offers distinct advantages. In older adults, depressive symptoms are known to fluctuate over time and be affected by seasons, weather, major life events, etc. These symptoms also show a natural tendency to remit spontaneously [35]. In such time trends are subsumed into the error term or are unbalanced across groups, they could confound the effect estimate. The core strength of the SW-CRT is that its sequential rollout of the intervention across multiple time points enables the modeling and statistical control of such underlying time trends, separating them from the true intervention effect.

To address these gaps, the Geriatric Exercise-Sleep Optimization (GESO) trial protocol was designed by adopting a SW-CRT design. The primary aim is to evaluate the clinical efficacy of an integrated exercise-sleep intervention for alleviating depressive symptoms among community-dwelling older adults, with a particular interest in those with subthreshold depression. Secondary aims are to explore potential underlying mechanisms of the intervention by assessing salivary biomarkers (BDNF and irisin) and EEG; and to evaluate the cost-effectiveness of the intervention from a public healthcare system perspective.

## Methods

### Design and settings

The GESO project is a pragmatic, open-labeled, stepped-wedge cluster randomized trial incorporating qualitative process and economic evaluations. A concise causal model diagram was showed in Supplementary 1. This study will be conducted in accordance with the SPIRIT (Standard Protocol Items: Recommendations for Interventional Trials) statement [36].

This study will be conducted in six communities in Urumqi City, Xinjiang Uygur Autonomous Region, China. Participating communities will be selected based on diverse geographic distribution, participant sociodemographic characteristics, and approval from local administrative committees. We will distribute flyers containing relevant information about our study to older residents, and invite them to attend their local community centers for assessment and the consent process.

### Participants and recruitment

Participants meeting the following criteria will be included: (1) aged 60 years or above; (2) current depressive symptoms as measured by the Patient Health Questionnaire-9 (PHQ-9) score ≥ 5. The exclusion criteria are: (1) cognitive impairment indicated by a score < 26 on the Montreal Cognitive Assessment (MoCA); (2) contraindications to exercise (e.g., uncontrolled hypertension or diabetes mellitus, unstable angina); (3) current participation in any other intervention program. All participants will provide written informed consent.

### Study procedure

All eligible participants will run over a 12-week integrated exercise and sleep intervention. A trained GESO research assistant will contact potential participants via face-to-face meeting, telephone call, or online platforms (e.g., voice call or video call) to introduce the study, discuss participation, answer questions, and determine eligibility. Then, eligible participants who express willingness to join will then provide a signed written informed consent. For participants contacted remotely (via telephone or online) will sign the informed consent form during the baseline assessment.

To minimize participant burden, we will deliberately distribute all assessments across the study period. Specifically, subjective measurements (i.e., questionnaires and scales) will be collected at the beginning of the first week. Then, researchers will equip with the ActiGraph accelerator device (GT3X) and give usage instructions for each participant. After a one-week monitoring period, participants will be required to return the device. The saliva sample and resting-state EEG will be collected within one week after allocation and post-intervention (12-week) according to participant availability. Notably, the EEG and saliva collection are part of an optional sub-study on mechanisms. For those who consent to this sub-study, we offer flexible scheduling and transportation assistance to facilitate participation. Participation is voluntary and does not affect involvement in the main trial or compensation. Other proactive strategies in order to enhance engagement and minimize dropout include a dedicate research assistant to maintain weekly contact, offering a highly flexible schedule, providing small, tiered compensation after each major time point, and giving personalized guidance and feedback on participants’ health conditions and improvements.

### Randomization and blinding

**Fig. 1:**
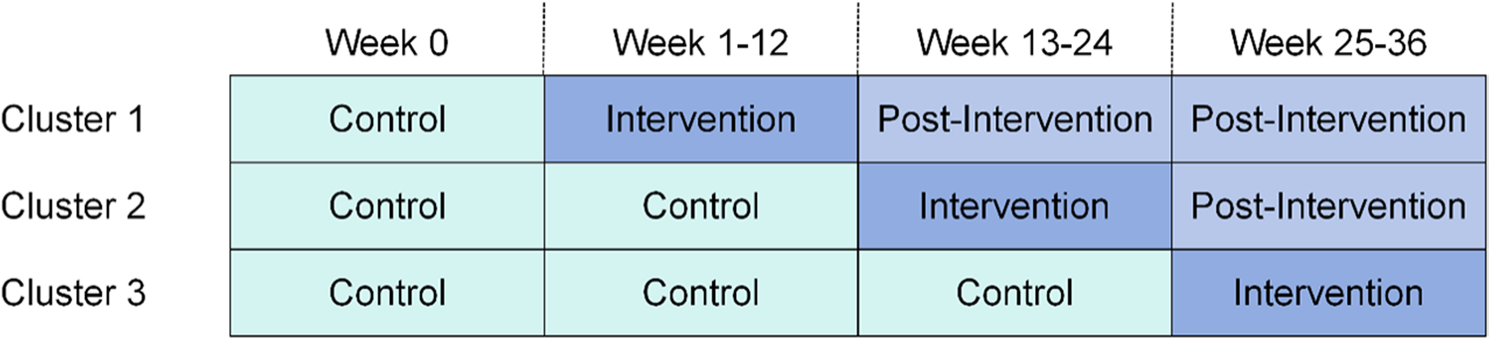
Randomization schedule

Initially, all six communities will begin in the control phase. Subsequently, two communities will be randomly allocated to the 12-week intervention period, followed by two other communities 12 weeks later, and the final two communities 12 weeks after that. Trial randomization will be performed by an independent researcher not otherwise involved in the study. Each community will be notified of its own transition date after recruiting first participant but will not be notified of the transition dates of other communities. Details of the randomization schedule are given in Fig. 1.

As this is an open-label study, both participants and intervention deliverers will be aware of allocation. Participants will be informed that they will receive a 12-week home-based exercise and sleep health intervention with mobile phone support. However, the participating communities will not be aware of the allocation sequences for themselves or others. We will also blind the assessors (data collectors) and statisticians to group allocation.

### Interventions

Based on the literature review [37–39] and our previous study, a preliminary integrated intervention framework was developed for older adults with depression. The intervention consists of two core components: a multi-component exercise and sleep hygiene education (SHE). These components are theoretically and practically synergistic and are delivered in a coordinated manner over the 12-week period, supported by behavioral activation (BA) techniques to facilitate adherence and behavior change [40, 41].

- Exercise component: The exercise program is primarily based on the Otago Exercise Program [42] and will be refined in consultation with sports experts. It is designed as three time-stratified regimens to align with circadian rhythms and daily routines:
  - Morning regimen (10 minutes): Focusing on low-intensity activation and circadian entrainment upon waking, involving dynamic stretching and light exposure.
  - Afternoon regimen (20 min): Constituting the core exercise session, including low to moderate intensity aerobic exercise, resistance training, and balance training. Exercise intensity will be standardized using the Borg Rating of Perceived Exertion (RPE 6–20) scale. Participants will be trained to maintain an RPE between 11 and 16 (low to moderate intensity) during the aerobic session [43, 44].
  - Pre-sleep regimen (15 min): Aiming at promoting physiological and cognitive relaxation within 1 h of bedtime, primarily consists of flexibility training. Participants will also be encouraged to incorporate brief ‘zero-time’ activities throughout the day to interrupt sedentary behavior or physical inactivity.
- Sleep component: The SHE is based on established sleep hygiene recommendations [45]. It comprises structured behavioral advice concerning modifiable lifestyle and environmental factors associated with sleep quality [11]. Key education topics include regulating sleep-wake schedules, optimizing the bedroom environment (e.g., reducing light and noise), scheduling napping, managing caffeine and alcohol intake, and establishing a pre-sleep wind-down routine.

The two components are integrated into a unified, home-based intervention package. BA techniques including goal setting, activity scheduling and monitoring, reinforcement, and relaxation training, will be incorporated into the 12-week intervention implementation to support the adoption and maintenance of the integrated exercise-sleep intervention. In the control phase, participants will be advised to maintain their normal daily activities and will only receive minimum contact (once per week) to reduce the pre-intervention attrition. The final integrated intervention will be refined through a preliminary test with 20 participants based on rationale about feasibility. Detailed description of the intervention, according to the template for intervention description and replication (TIDieR) checklist [46], is provided in Supplementary 2.

### Data management plan

Data will be securely stored on offline computers and in secure locations. Access to participant data will be restricted according to researcher role. Participant confidentiality will be maintained at all times except in cases where significant risk of harm to self or others is identified.

### Sample size

Power calculation was performed using the PASS software module ‘Tests for Two Means in a Stepped Wedge Cluster Randomized Design’. Key input parameters were derived from a similar previous study [47], which reported a mean reduction in depressive symptom of 4.0 in intervention group compared to 2.0 in control group among community-dwelling older adults with depression. Based on our stepped-wedge design with 6 clusters, 2 clusters crossing over per sequence, an intra-cluster correlation (ICC) of 0.1, a planned sample size of 22 per community, a desired power of 90%, a two-side α level of 0.05, and an estimated 20% dropout rate, a total sample size of 165 participants will be required.

### Measurement of outcomes

The primary outcome will be self-reported depressive symptoms and remission rates, as measured by the PHQ-9 at 12-week post-intervention. Depression remission is defined as a PHQ-9 score < 5. The PHQ-9 is more specific for detecting depression in older adults [48] and is recommended for depression screening and severity assessment by the Chinese Society of General Practice, the Depressive Disorder Collaboration Group of Chinese Society of Psychiatry, the US Preventive Services Task Force, and others [17, 49, 50]. The consistency rate between PHQ-9 and Geriatric Depression Scale-15 (GDS-15) is 96.10% [51].

Secondary outcomes are additional health outcomes, cost-effectiveness, and potential mechanisms. Additional health outcomes include quality of life (measured by the World Health Organization Quality of Life-Older Adults, WHOQOL-OLD), physical activity levels, daily step counts, subjective sleep quality (measured by the Pittsburgh Sleep Quality Index, PSQI), objective sleep parameters including total sleep time (TST), wake after sleep onset (WASO), sleep onset latency (SOL), and sleep efficiency (SE), and anxiety symptom (measured by the Generalized Anxiety Disorder 7-item scale, GAD-7). Objective outcomes of physical activity levels, daily step counts, and objective sleep parameters will be obtained using a wrist-worn ActiGraph accelerometer for 7 consecutive days. This approach has been demonstrated to be effective among older adults in previous studies [52, 53].

For cost-effectiveness analysis, costs will be aggregated across all clusters using a bottom-up approach, and calculated individually for each cluster in the trial. The cost data for the economic evaluation will be collected from study records (e.g., personnel time, equipment, materials) and does not impose any additional data collection burden on the participants themselves. The economic evaluation will be conducted from a public healthcare system perspective. We plan to develop an intervention cost form for this purpose that covers and sums up all GESO-intervention costs and payments that would be reimbursed by health insurance companies if the respective intervention will be accepted for application in routine health care. All costs will be valued in Chinese Yuan (CNY), with prices adjusted to the 2024 value level. The cost-elements (e.g. wages of trainers, session or room cost, seminar documents, program development etc.) included into the calculation varied considerably depending on the various study-interventions or -programs. Cost of professional support will be estimated from relevant salary scales and published reports/literature. As the economic end point for the cost-utility analysis, we will consider the cost per quality-adjusted life year (QALY) gained. We will derive QALYs from the Five-level EuroQol Five-dimensional Questionnaire (EQ-5D-5 L) from T0 to T2 [54] using the norms for the urban Chinese population proposed by Yang [55]. In accordance with the China Guidelines for Pharmacoeconomic Evaluations (2020), we will discount both future costs and health outcomes (QALYs) at an annual rate of 5% for the base-case analysis, and employ discount rates ranging from 0% to 8% in sensitivity analyses to test the robustness of the results [56].

Saliva samples for the analysis of BDNF and irisin will be collected at baseline and at 12-week for biochemical assessments. To avoid the influence of circadian variation, sample collection will take place at the same time of the day, between 10:00 a.m. to 12:00 p.m. Participants will be asked to avoid food intake and oral chewing to prevent their effects on sample concentration. Before sample collection, participants will rinse their mouth with clean water and then drool into a 50mL enzyme-free centrifuge tube. Saliva samples will be centrifuged immediately at 10,000 rpm for 2 min, then, the supernatant will be collected and stored at −80℃. Saliva BDNF and irisin levels will be quantified by enzyme-linked immunosorbent assay (ELISA) using specific kits (BDNF kit: Cat. E-EL-H0010; Irisin kit: Cat. E-EL-H5735, Elabscience) following the manufacturer instructions.

EEG is a non-invasive, economical method to explore the electrocortical brain activity, and also has been applied to capture functional activity in interventional studies [57, 58]. Resting-state EEG data will be collected at baseline (T0) and post-intervention (T2) for exploring the neurophysiological mechanisms of the intervention. To facilitate participation and minimize burden, we will offer all participants flexible scheduling, transportation assistance or reimbursement to the lab, and a guided, relaxed assessment experience in a dedicated facility. EEG measurements will take place in a dimly lit room within the School of Psychology at Xinjiang Normal University. Participants will sit in a relaxed position, with their backs supported by the chair and both hands comfortably resting on a table in front of them. They will be instructed to perform an average of 12 min (10 to 15 min) eye-closed resting-state task followed by a 3-minute eyes-open resting condition. Data will be acquired using an ANT amplifier (Compumedics Neuroscan, SynAmps2,) equipped with a 64-channel active electrode cap arranged according to the international 10–20 system. Three electrooculogram (EOG) channels will be used to detect ocular artifacts. All signals will be referenced online to Cz and grounded at AFz, and digitized at a sampling frequency of 500 Hz. Electrode impedances will be maintained below 10 kΩ. Data will be processed and analysed with MATLAB R2024a (MathWorks, Natick, MA) in conjunction with the EEGLAB toolbox (v.2025.0.0) and custom-written scripts. All signals will be filtered using a 1 Hz high-pass filter, 80 Hz low-pass filter, and 50 Hz notch filter, then be down-sampled to 500 Hz.

Based on previous exploratory neurophysiological studies in older populations, we anticipated a good participation rate in this assessment. The resulting sample size for EEG analysis is expected to be sufficient for generating preliminary hypotheses regarding intervention-related neurophysiological changes, consistent with similar exploratory mechanistic studies. Study enrolment and assessment procedures are described in Table 1.

**Table 1:** Schedule of enrolment and assessments

Timepoint	-T1	T0	T1	T2
	Enrolment and allocation	Baseline	6-week	12-week
Enrolment				
Eligibility screen	×			
Informed consent	×			
Allocation	×			
Assessment				
Demographic information		×		
Primary outcomes				
Depressive symptom: PHQ-9		×	×	×
Depression remission rate		×	×	×
Secondary outcomes				
Quality of life: WHOQOL-OLD		×		×
Physical activity: ActiGraph accelerometer measured levels and daily step counts		×		×
Subjective sleep quality: PSQI		×	×	×
Objective sleep: ActiGraph accelerometer measured TST, WASO, SOL, and SE		×		×
Anxiety symptom: GAD-7		×	×	×
Economic evaluation				
Costs				×
QALY: EQ-5D-5 L		×		×
Saliva collection (BDNF, irisin)		×		×
EEG		×		×

### Process evaluation

Consistent with Medical Research Council (MRC) guidance for trials of complex interventions, we will conduct a process evaluation [59]. This evaluation aims to explore the fidelity of implementation, mechanisms of impact, and contextual factors that facilitate or impede intervention delivery. We will pay particular attention to documenting and analyzing contextual variations across clusters (e.g., socio-cultural characteristics, access to health services). This will complement the outcomes evaluation through exploring: (1) fidelity of intervention delivery by different researchers; (2) participants satisfaction with treatment; (3) adherence rates; (4) dropout rates; (5) participants’ experience of the integrated intervention, including perceived benefits, facilitators, barriers, and any unexpected consequences; and (6) identify contextual factors across different clusters and explore the role in shaping the implementation process of intervention.

### Statistical analyses

Analyses will include descriptive summaries and preliminary unadjusted comparisons between phases (control vs. intervention) and clusters. Specifically, variables within each phase or clusters will be described using descriptive statistics (means and 95% confidence intervals for normal distributed quantitative variables; medians and interquartile ranges for non-normally distributed quantitative variables).

Analyses of data from a SW-CRT requires specific methodological considerations to account for its design, including the control of secular trends (period effects) and the assessment of potential carryover effects. Guided by methodological recommendations for SW-CRTs [60], we will choose Method 3 to evaluate the intervention effects of the integrated exercise-sleep intervention. This method treats the intervention variable as a time-dependent categorical variable, comparing groups with different numbers of intervention measurements with all the control measurements. This allows us to examine whether the effect of the integrated exercise–sleep intervention varies with the length of exposure, thereby informing the time dependency of treatment effects, including potential carryover.

All models will be estimated using multilevel mixed-effects regression to account for the hierarchical structure of the data. We will first fit a two-level (time and individual levels) and a three-level (time, individual, and community levels) mixed-effects model without any potential confounders. Then, we will further adjust the selected model (based on model fit criteria) by including study period (time) as a fixed effect. Third, an adjustment will be made for the baseline value of the outcome variable and associated baseline covariates (e.g., socio-demographics, cognition), and in the fourth step, both an adjustment for the follow-up time, the baseline value of the outcome variable, and related covariates will be performed. To evaluate the time dependency of the effect of starting the intervention, an interaction term between intervention and time can be added to the model [60, 61]. Additionally, we will account for potential between-cluster variations by including relevant community-level covariates (e.g., socio-cultural characteristics and access to health services) as fixed effects in the models, if they show preliminary associations with the outcome.

The economic evaluation will include both cost-effectiveness analysis (CEA) and cost-utility analysis (CUA), considering only costs and effects occurring within the trial timeframe. CEA will assess the intervention effect on depression symptoms relative to costs in older adults. Results will be expressed as cost-effectiveness ratio (CER) and incremental cost-effectiveness ratio (ICER). CUA will be calculated by QALYs and cost-utility ratio (CUR). For the exploratory analyses of mechanistic outcomes, we will examine the intervention effects on the biomarkers (i.e., salivary BDNF and irisin levels) from baseline to post-intervention. Analyses of EEG data will be conducted on the subset of participants who complete this assessment and are therefore exploratory in nature. We will compare baseline characteristics between participants who do and do not complete the EEG to assess potential selection bias. Indicators including absolute power (AP), phase locking value (PLV), phase lag index (PLI), and weighted phase lag index (WPLI) will be analyzed to investigate potential neurophysiological mechanisms underlying the intervention’s effectiveness.

Primary analysis will follow the intention-to-treat (ITT) principle, including all randomized participants. Primary and secondary outcomes will be performed with generalized linear mixed models (GLMMs). Estimated intervention effects will be reported as mean differences for continuous outcomes or odds ratio for binary outcomes between intervention and control periods. To handle missing outcome data, we will employ multiple imputation by chained equations (MICE) under the assumption of missing at random. Sensitivity analyses will be conducted to assess the robustness of the findings by: (1) comparing results from the ITT analysis with those from a per-protocol (PP) analysis; (2) evaluating the impact of missing data under different assumptions (e.g., missing not at random scenarios); (3) adjusting for baseline depression symptom severity; and (4) using different depression classifications (i.e., the total score of PHQ-9 or the diagnostic criteria for depression based on DSM-IV [30, 62]). All analysis will be performed by an independent statistician using R software version 4.3.2 (R Core Team, R Foundation for Statistical Computing, Vienna, Austria, https://www.R-project.org). Statistical significance will be defined as a two-tailed *p*-value < 0.05.

### Ethical approval and protocol registration

This study was approved by the Ethics Review Committee of Xiangya School of Nursing, Central South University (No. E2025144). The trial protocol was registered on www.chictr.org.cn (Registration number: ChiCTR2500107641) prior to the recruitment of the first participant. The study will be conducted in accordance with ethical principles, including obtaining written informed consent from all participants during the baseline assessment. All researchers have signed confidentiality agreements. Upon completion of study participation, participants will receive an acknowledgment letter and 100 CNY as compensation for their time.

## Discussion

Both empirical evidence and theoretical underpinnings underscore the significant roles of exercise and sleep health in maintaining physical and psychological health. Targeting both should alleviate depressive symptoms, improve quality of life and physical health. The GESO project therefore aims to evaluate the effectiveness and cost-effectiveness of a home-based, integrated exercise-sleep intervention for depressed community-dwelling older adults using a SW-CRT study design.

To our best knowledge, this is the first study incorporating a cost-effectiveness analysis of an integrated exercise-sleep intervention for this population. Several lines of results are anticipated from this study. Our intervention was tailored for depressed older adults through a multi-step process involving cognitive interviewing, pilot testing, and expert consultation, incorporating identified facilitators and barriers. Regarding the delivery format, this study evaluates a home-based integrated intervention. This contrasts with most RCTs where the training is typically conducted in group sessions under the supervision of an instructor outside the home environment. The home-based format enables researchers to provide extensive expert-validated online resources (text, images, videos), and is potentially more cost-effective. For outcome evaluation, cost-effectiveness analysis and cost-utility analysis will be conducted from a public health service perspective. These analyses will highlight the intervention’s significance and assess its potential for scalability. Regarding mechanisms of effectiveness, we plan to explore the underlying mechanisms by analyzing EEG-derived functional connectivity along with salivary levels of BDNF and irisin.

There are some limitations in this study. First, although the proposed SW-CRT design has several advantages over parallel group RCTs, it is not as methodologically rigorous as a true cluster-randomized design, and inevitably brings challenges in recruitment and implementation [63], possibly create time contamination and complexity of analysis. Nevertheless, recruitment of the GESO project will be community-based participatory research, that is, community organizations will contribute to both the recruitment and implementation phases of the project through activities such as promotion and participant mobilization, assistance with follow-ups, and the provision of venue resources. Second, this study design precludes the effectiveness evaluation of the core active components or optimal strategies compare with overall intervention package. For instance, it cannot determine the optimal sequence for delivering exercise versus sleep intervention or evaluate the potential of alternative delivery agents (e.g., virtual agents, primary care nurses, non-specialist assistants). Future studies could employ a sequential multiple assignment randomized trial (SMART) design to optimize strategies and test such alternative agents. Third, the collection of EEG data is optional and require additional time (e.g., commuting time) for participants, which is likely to result in a subsample of participants with greater motivation or better physical mobility. Therefore, the findings of EEG should be interpreted as preliminary and may not generalize to the full study population. Finally, culturally and linguistically diverse residents are likely to be underrepresented, due to constraints in financial and human resources, which may limit the generalizability of the results. Further multicenter studies recruiting a wider range of older adults with larger sample sizes are recommended.

In conclusion, the present study will advance the extant knowledge about effectiveness, acceptability, and cost-effectiveness of an integrated exercise-sleep intervention, with a specific focus on older adults with depression in community settings. The findings will contribute to providing a more cost-effective, evidence-based, and scalable lifestyle intervention for this population.

### Data Availability

The datasets generated during the present study can be obtained from the corresponding author on reasonable request, respecting the participant confidentiality. The trial results will be communicated via publications.

## Supplementary Information

Supplementary Material 1.

## Data Availability

The datasets generated during the present study can be obtained from the corresponding author on reasonable request, respecting the participant confidentiality. The trial results will be communicated via publications.
