# Supplementary material for: Effect of a digital-based integrated exercise and sleep intervention for older adults with depression: study protocol for a stepped-wedge cluster randomized controlled trial
Source: BMC Geriatr. 2026 Feb 2;26:285. doi: 10.1186/s12877-026-07071-z (PMC12952190; doi:10.1186/s12877-026-07071-z)
Supplement: Supplementary file 1 — Supplementary Material 1. [file 12877_2026_7071_MOESM1_ESM.docx]

Supplementary 1. Causal model diagram of the GESO project

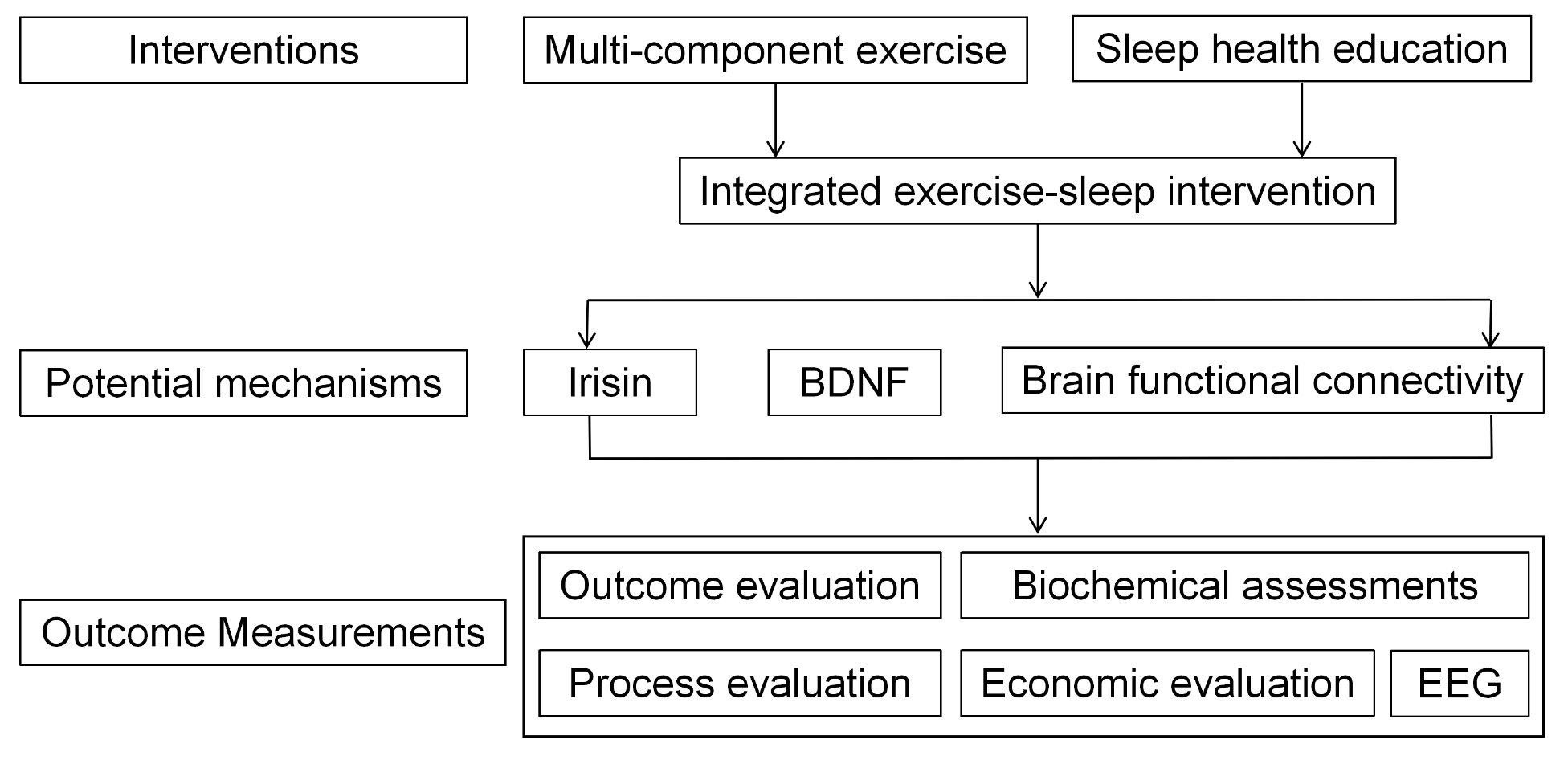


Supplementary 2. The integrated exercise and sleep health intervention (GESO project) 12-weeek agenda

| Session (Week) | | Goal | Core of interventions | Recommended form |
| --- | --- | --- | --- | --- |
| 1 | Introduction: Increase awareness of the need to exercise and sleep promotion | | 1. Self-introduction and sign the informed consent form  2. Introduce mental health benefits of exercise-sleep interventions through a video produced by the research team | Offline (in-person) |
| 2 | Core: Establish **Morning** routine | | 1. Create morning exercise plan in collaboration with participants  2. Emphasize the importance of get up on time and morning light exposure for sleep quality  3. Emphasize the importance of self-monitoring and record-keeping  4. Weekly encouragement via message | Online (in-person) |
| 3 | Core: Establish **Afternoon** routine | | 1. Review morning activity compliance and problem-solving  2. Create afternoon activity plan in collaboration with participants  3. Discuss the impact of napping: advise to avoid long or late naps  4. Weekly encouragement via message | Online (in-person) |
| 4 | Core: Establish **Evening** routine (Pre-sleep) | | 1. Review morning and afternoon activity compliance and problem-solving  2. Create evening activity plan in collaboration with participants  3. Optimize the sleep environment (e.g., dark, quiet bedroom)  4. Weekly encouragement via message and sleep hygiene tips | Online (in-person) |
| 5 | Core: Full-day routine adherence | | 1. Review full-day activity compliance and problem-solving  2. Barrier-coping training (e.g., modified exercise for fatigue)  3. Weekly encouragement via message and sleep hygiene tips | Online (in-person) |
| 6 | Core: Full-day routine adherence | | 1. Review full-day activity compliance  2. Problem-solving via telephone/video call, focusing on specific sleep or exercise challenges  3. Weekly encouragement via message | Online (in-person) |
| 7 | Supportive: Maintenance | | 1. Weekly encouragement via message  2. Problem-solving via telephone/video call  3. Introduce and practice a relaxation technique to facilitate sleep onset (e.g., diaphragmatic breathing or progressive muscle relaxation) | Online (in-person) |
| 8 | Core: Exercise Progression | | 1. Encourage and establish tailored exercise progression (e.g., increase the weekly frequency, enhance exercise intensity through adding 2-3 sets to resistance training sessions, or extending duration of single-session by 5-10 minutes)  2. Discuss how exercise can deepen sleep and how good sleep aids exercise recovery. Provide guidance on timing of exercise to avoid sleep disruption  3. Weekly encouragement via message | Online (in-person) |
| 9 | Supportive: Maintenance | | 1. Weekly encouragement via message  2. Problem-solving focused on managing common age-related sleep challenges (e.g., pain, nocturia, advanced sleep phase) via telephone/video call | Online (in-person) |
| 10 | Core: Exercise Progression | | 1. Encourage further tailored exercise progression  2. Review and identify improvements and lingering issues, reinforce strategies that are working well  3. Weekly encouragement via message | Online (in-person) |
| 11 | Supportive: Maintenance | | 1. Weekly encouragement via message  2. Problem-solving focused on planning for future challenges (e.g., holidays, travel, illness) to maintain routines via telephone/video call | Online (in-person) |
| 12 | Summary: Conclusion and sustainability | | 1. Individual progress review of both exercise and sleep outcomes  2. Develop personalized sustainable behavior strategies: identify individual/environmental risk factors, and establish long-term maintenance plans | Offline (in-person) |
